# Apixaban versus Antiplatelet drugs or no antithrombotic drugs after anticoagulation-associated intraCerebral HaEmorrhage in patients with Atrial Fibrillation (APACHE-AF): study protocol for a randomised controlled trial

**DOI:** 10.1186/s13063-015-0898-4

**Published:** 2015-09-04

**Authors:** Koen M. van Nieuwenhuizen, H. Bart van der Worp, Ale Algra, L. Jaap Kappelle, Gabriel J. E. Rinkel, Isabelle C. van Gelder, Roger E. G. Schutgens, Catharina J. M. Klijn, on behalf of the APACHE-AF investigators

**Affiliations:** Department of Neurology and Neurosurgery, Brain Center Rudolf Magnus, University Medical Center Utrecht, G03.232, PO Box 85500, 3508 GA Utrecht, The Netherlands; Julius Center for Health Sciences and Primary Care, University Medical Center Utrecht, STR. 7.140, PO Box 85500, 3508 GA Utrecht, The Netherlands; Department of Cardiology, University Medical Center Groningen, PO Box 30.001, 9700 RB Groningen, The Netherlands; Van Creveldkliniek, University Medical Center Utrecht, C01.425, PO Box 85500, 3508 GA Utrecht, The Netherlands; Department of Neurology, Radboud University Medical Center, PO Box 9101, 6500 HB Nijmegen, The Netherlands

**Keywords:** Antiplatelet drugs, Apixaban, Atrial fibrillation, Intracerebral haemorrhage, Randomised controlled trial

## Abstract

**Background:**

There is a marked lack of evidence on the optimal prevention of ischaemic stroke and other thromboembolic events in patients with non-valvular atrial fibrillation and a recent intracerebral haemorrhage during treatment with oral anticoagulation. These patients are currently treated with oral anticoagulants, antiplatelet drugs, or no antithrombotic treatment, depending on personal and institutional preferences.

Compared with warfarin, the direct oral anticoagulant apixaban reduces the risk of stroke or systemic embolism, intracranial haemorrhage, and case fatality in patients with atrial fibrillation. Compared with aspirin, apixaban reduces the risk of stroke or systemic embolism in patients with atrial fibrillation, and has a similar risk of intracerebral haemorrhage. Novel oral anticoagulants have not been evaluated in patients with atrial fibrillation and a recent intracerebral haemorrhage.

To inform a phase III trial, the phase II Apixaban versus Antiplatelet drugs or no antithrombotic drugs after anticoagulation-associated intraCerebral HaEmorrhage in patients with Atrial Fibrillation (APACHE-AF) trial aims to obtain estimates of the rates of vascular death or non-fatal stroke in patients with atrial fibrillation and a recent anticoagulation-associated intracerebral haemorrhage treated with apixaban and in those in whom oral anticoagulation is avoided.

**Methods/Design:**

APACHE-AF is a phase II, multicentre, open-label, parallel-group, randomised clinical trial with masked outcome assessment. One hundred adults with a history of atrial fibrillation and a recent intracerebral haemorrhage during treatment with anticoagulation in whom clinical equipoise exists on the optimal stroke prevention strategy will be enrolled in 14 hospitals in The Netherlands.

These patients will be randomly assigned in a 1:1 ratio to either apixaban or to avoiding oral anticoagulation. Patients in the control group may be treated with antiplatelet drugs at the discretion of the treating physician. The primary outcome is the composite of vascular death or non-fatal stroke during follow-up. We aim to include 100 patients in 2.5 years. All patients will be followed-up for the duration of the study, but at least for 1 year. Recruitment commenced in September 2014 and is ongoing. This trial is funded by the Dutch Heart Foundation (2012 T077) and ZonMW (015008048).

**Trial registration:**

NTR4526 (16 April 2014).

**Electronic supplementary material:**

The online version of this article (doi:10.1186/s13063-015-0898-4) contains supplementary material, which is available to authorized users.

## Background

Stroke is a major cause of death and disability and is associated with high healthcare expenditure [1, 2]. About 80 % of strokes are ischaemic, 15 % intracerebral haemorrhage (ICH), and 5 % subarachnoid haemorrhage [3].

Cardioembolism, most often caused by non-valvular atrial fibrillation (AF), accounts for 13 to 27 % [4, 5] of ischaemic strokes. The average annual risk of ischaemic stroke in patients with AF not treated with antithrombotic drugs is 4.5 % [6]. The risk of ischaemic stroke can be estimated with the CHA_2_DS_2_-VASc score [7]. The annual thromboembolic event rate increases from 2.2 % with a CHA_2_DS_2_-VASc score of 2 to 15.2 % with the maximum score of 9 [8]. In patients with AF and a CHA_2_DS_2_-VASc score ≥ 1, treatment with an oral anticoagulant (OAC) is recommended [9, 10]. Traditionally, vitamin K antagonists (VKA) have been the first choice for prevention of thromboembolic events in these patients. While VKA therapy decreases the risk of ischaemic stroke, it increases the risk of ICH, the most devastating complication of OAC therapy. The risk of ICH in patients with AF treated with vitamin K antagonists is 0.3 to 3 % per year, and increases with increasing age [11–14]. This complication leads to death in 45 % of cases, and only 17 % of patients recover without disability [15].

In patients with AF who are unsuitable for VKA therapy because of poor compliance or allergy, or who are unwilling to receive this therapy, antiplatelet drugs (APDs, e.g. acetylsalicylic acid, clopidogrel) may be considered [9, 10]. However, antiplatelet treatment only results in a modest reduction in the risk of ischaemic stroke [16] and increases the risk of major bleeding as compared to no antithrombotic therapy (incidence rate ratio 1.55; 95 % confidence interval (CI), 1.48–1.63)) [17].

In patients with AF who survive an anticoagulation-related ICH, a longstanding and pressing clinical dilemma is whether or not to resume treatment with oral anticoagulation to prevent ischaemic stroke and other future thrombotic and embolic complications [18, 19]. Randomised trials have not been performed and reliable estimates of the risk of recurrent ICH or ischaemic stroke after resumption of antithrombotic drugs on the one hand, or permanent discontinuation of these drugs on the other, are lacking [20].

Patients with AF on warfarin who survive an ICH have a higher event rate for the combination of ischaemic stroke, systemic embolism or transient ischaemic attack in the 2 years following the ICH compared with AF patients on warfarin who did not experience an ICH (event rate ratio, 5.40 (95 % CI 4.04 to 7.22) [21].

In retrospective studies of small patient cohorts, the annual risk of recurrent ICH after resumption of VKAs varied between 2.5 and 20 % and that of ischaemic stroke between 0 and 33 % [22–26]. In patients in whom VKAs were discontinued permanently, the risk of ICH varied between 0 and 9 % and the risk of ischaemic stroke between 10 and 48 % [22–26].

In small observational studies of patients who survived an ICH – regardless of whether this occurred during the use of a VKA – no difference was found in the risk of recurrent ICH between patients treated with acetylsalicylic acid after the initial ICH and those who used no antithrombotic drugs [25, 27–29], with an annual risk of recurrent ICH between 2.3 and 8.2 % [27, 29]. The annual risk of ischaemic stroke in these patients varied between 1.3 and 9.4 %.

The above-mentioned estimates for the risk of ischaemic stroke or recurrent ICH during treatment with VKA or APD are not reliable because of selection bias, the inclusion of patients with different indications for the use of antithrombotic drugs, and variation in target international normalised ratios (INRs). In addition, estimates varied widely between studies and had wide CIs because of the small numbers of patients included.

The aforementioned information illustrates that there is a need for evidence-based recommendations for the prevention of stroke and other thromboembolic complications in survivors of anticoagulation-related ICH [30, 31]. Currently, patients are treated – based on ‘expert opinion’ – with OAC, APDs, or no antithrombotic medication at all, resulting in marked practice variation [32].

In the past few years, novel, direct anticoagulant drugs (DOACs) have been introduced in clinical practice. These drugs reduce coagulation by inhibition of factor Xa (rivaroxaban, apixaban and edoxaban) or factor IIa (dabigatran), resulting in a reduced thrombin generation, diminished enzymatic conversion of fibrinogen to fibrin and, thus, less efficient clot formation.

In the randomised Apixaban for Reduction in Stroke and Other Thromboembolic Events in Atrial Fibrillation (ARISTOTLE) study, the oral factor Xa inhibitor apixaban at a dose of 5 mg twice daily was more effective than warfarin (target INR, 2.0 to 3.0) in preventing stroke or systemic thromboembolism in patients with AF: hazard ratio, 0.79; 95 % CI, 0.66 to 0.95; *P* < 0.001 for non-inferiority; *P* = 0.01 for superiority. Patients treated with apixaban less often had an ICH (hazard ratio, 0.51; 95 % CI, 0.35 to 0.75; *P* < 0.001) than patients treated with warfarin [33]. These beneficial effects were seen throughout different times in therapeutic range (TTR) ranges [34].

Of the DOACs, only apixaban has been compared with acetylsalicylic acid in a randomised controlled trial in patients with AF. In the trial Apixaban Versus Acetylsalicylic Acid to Prevent Stroke in Atrial Fibrillation Patients Who Have Failed or Are Unsuitable for Vitamin K Antagonist Treatment (AVERROES), patients with AF who were treated with apixaban 5 mg twice daily had a lower risk of stroke or systemic embolism than patients treated with acetylsalicylic acid at a dose of 81 to 324 mg per day (hazard ratio 0.45; 95 % CI 0.32 to 0.62; *P* < 0.001), whereas the rates of ICH in the 2 groups were similar [35].

In phase III randomised trials comparing other DOACs with warfarin in patients with AF, these were non-inferior to warfarin in the prevention of stroke and systemic embolism and were associated with a reduced risk of intracranial bleeding [36–38].

In a meta-analysis of phase III randomised trials of patients with AF who were randomised to receive DOACs or warfarin, the DOACs had a favourable risk-benefit profile, with significant reductions in stroke, intracranial haemorrhage, and mortality, and with a similar major bleeding risk as for warfarin, but an increased risk of gastrointestinal bleeding. The relative efficacy and safety of DOACs was consistent across a wide range of patients [39].

The DOACs have not been compared against each other in clinical trials. A meta-analysis using a Baysian random effects model suggested that the risk reductions for ICH as compared to warfarin are similar [40]. There are no clinical trials testing the effect of a DOAC in patients with AF and a recent oral anticoagulant-associated intracerebral haemorrhage (OAC-ICH).

We hypothesise that in patients with AF who survived an anticoagulation-associated ICH, treatment with apixaban may be the best long-term alternative for the prevention of recurrent stroke and systemic thromboembolism. To test this hypothesis, a conclusive phase III, randomised clinical trial comparing the long-term effects of apixaban with those of APDs or no antithrombotic treatment in these patients is required. Before such a trial can commence, a phase II trial is needed to obtain reliable estimates of the rates of vascular death or non-fatal stroke for both strategies in patients with AF and a recent anticoagulation-associated ICH. As a secondary objective, we aim to compare the rates of all-cause death, vascular death, stroke, ischaemic stroke, recurrent ICH, other major haemorrhage, systemic embolism, myocardial infarction, and functional outcome between patients treated with apixaban and those in whom anticoagulation is avoided.

### Rationale for study treatment

The treating physician will decide on any of the treatment regimens in the comparator group. This design is based on the lack of evidence that any of the treatment options has a more favourable risk-benefit ratio in this population than others [18, 32, 41, 42].

Allowing variable treatment regimens enables us to include patients with or without a medical history of atherosclerotic disease, which could warrant the use of APDs. The choice for these treatment options in the comparator arm enables us to achieve close similarity with current clinical practice and include as many eligible patients as possible to answer the research question.

The physician can include other indications for APDs (e.g., a history of myocardial infarction) in the decision on the treatment in the comparator arm.

Treatment with any of the drugs in the study can commence anywhere between 7 and 90 days after the ICH, at the discretion of the treating physician. There are reports suggesting that VKAs can be resumed after 3 days [43], whereas others recommend resumption of antithrombotic drugs anywhere between 70 and 210 days, if at all [24]. In the absence of evidence on the optimal timing of the resumption of antithrombotic drugs after ICH, we chose this interval as it reflects clinical practice.

## Methods/Design

### Overview

**Fig. 1:**
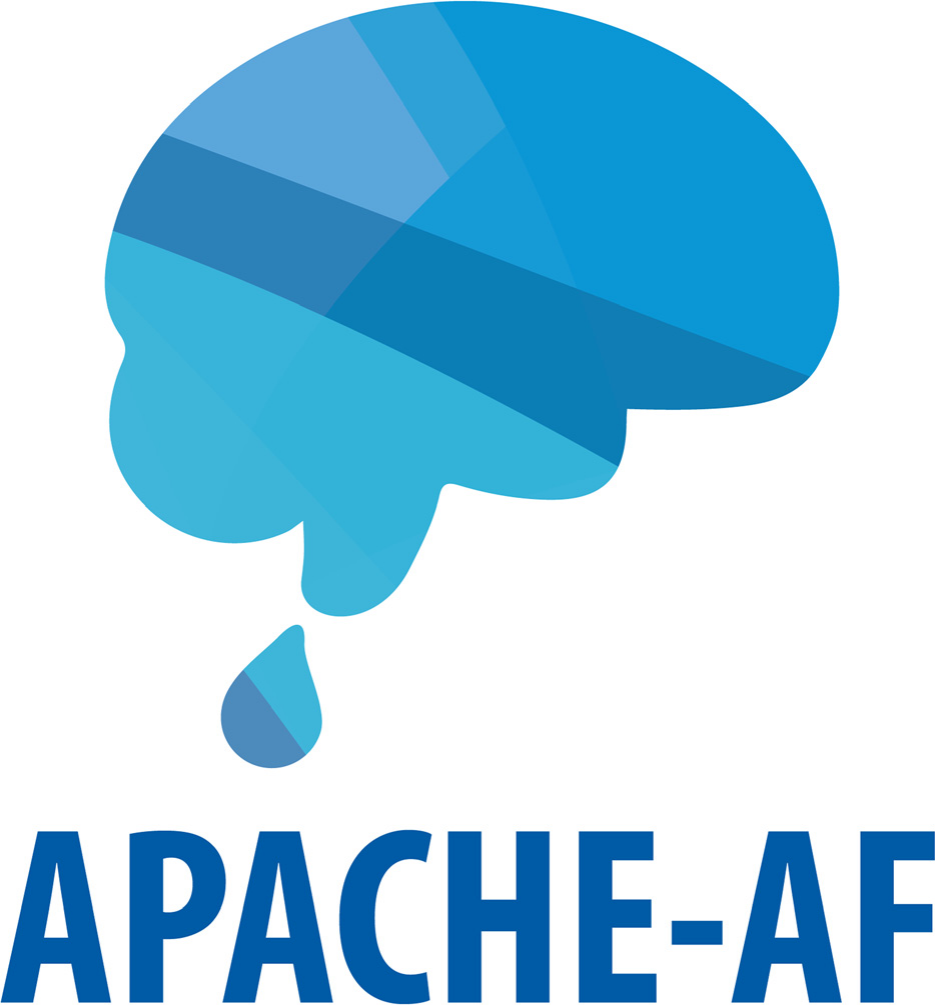
Trial logo

APACHE-AF (Fig. 1, http://www.apache-af.nl) is a phase II, randomised, open-label, parallel-group, multicentre clinical trial with masked outcome assessment (PROBE design [44]), comparing apixaban and avoiding anticoagulation in patients with AF and a recent anticoagulation-associated ICH. An adjudication committee blinded to treatment allocation will adjudicate outcomes.

A total of 100 patients will be included in 7 academic and 8 regional hospitals in the Netherlands over a period of 2.5 years. Members of the various study committees are listed in Appendix 1. Follow-up will continue until 1 year after inclusion of the last patient. The total study period is expected to be 4 years. Patient recruitment has started in September 2014.

### Eligible patients

Patients with AF who recently had an ICH during the use of anticoagulation, in whom there is clinical equipoise regarding the optimal medical treatment for the prevention of stroke, are eligible for participation in this trial. The ICH should have occurred between 7 and 90 days prior to randomisation. The CHA_2_DS_2_-VASc score should be 3 or higher [8] and the score on the modified Rankin Scale (mRS) [45], as a measure for disability, 4 or lower. Detailed eligibility criteria are shown in Table 1.

**Table 1:** Eligibility criteria

**Inclusion criteria**
Intracerebral haemorrhage (including isolated spontaneous intraventricular haemorrhage), documented with CT or MRI, during treatment with anticoagulation (VKA, any direct thrombin inhibitor, any factor Xa inhibitor, or (low-molecular-weight) heparin at a therapeutic dose)
The haemorrhage has occurred between 7 and 90 days before randomisation
Diagnosis of (paroxysmal) non-valvular AF, documented on electrocardiography
A CHA_2_DS_2_-VASc score ≥ 3
Score on the modified Rankin Scale (mRS) [45]
Equipoise regarding the optimal medical treatment for the prevention of stroke. The clinical equipoise should be self-reported by the attending neurologist after reviewing all relevant information available for the individual patient
Age ≥ 18 years
Written informed consent by the patient or by a legal representative
**Exclusion criteria**
Conditions other than AF for which the patient requires long-term anticoagulation
A different clinical indication for the use of an antiplatelet drug even if treated with apixaban, such as clopidogrel for recent coronary stenting
Mechanical prosthetic heart valve (biological prosthetic heart valves are allowed) or rheumatic mitral valve disease
Serious bleeding event in the previous 6 months, except for intracerebral haemorrhage^a^
High risk of bleeding (e.g. active peptic ulcer disease, a platelet count of < 100,000 per ml or haemoglobin level of < 6.2 mmol L^-1^ ischaemic stroke in the previous 7 days (patients are eligible thereafter), documented haemorrhagic tendencies, or blood dyscrasias)
Current alcohol or drug abuse
Life expectancy of less than 1 year
Severe renal insufficiency (a serum creatinine level of more than 221 μmol L^-1^ or a calculated creatinine clearance of < 15 ml per minute)
Alanine aminotransferase or aspartate aminotransferase level greater than twice the upper limit of the normal range or a total bilirubin more than 1.5 times the upper limit of the normal range, unless a benign causative factor (e.g. Gilbert’s syndrome) is known or identified
Allergy to apixaban
Use of strong cytochrome P450 3A4 (CYP3A4) and P-glycoprotein (P-gp) inhibitors (e.g. systemic azole-antimycotics such as ketoconazole or HIV protease inhibitors such as ritonavir)
Pregnancy or breastfeeding
Women of childbearing potential: any woman who has begun menstruation and is not postmenopausal or otherwise permanently unable to conceive. A postmenopausal woman is defined as a woman who is over the age of 45 and has not had a menstrual period for at least 12 months

^a^Serious bleeding event: see major extracranial haemorrhage and clinically relevant non-major bleeding in Table 3

*AF* atrial fibrillation, *CT* computed tomography, *MRI* magnetic resonance imaging, *VKA* vitamin K antagonist

#### Randomisation

Allocation to treatment groups will be based on randomisation through a web-based system. Treatment allocation will be stratified by the choice of treatment in the comparator arm (APD versus no APD) and will use a minimisation algorithm for age (≤75 years versus >75 years) and location of the haemorrhage (lobar versus non-lobar). This treatment allocation ensures that both treatment groups will be comparable with regard to the intended treatment in the comparator arm.

### Intervention

Patients will be randomised to treatment with apixaban 5 mg or 2.5 mg given orally twice daily, or to treatment with one or two oral APDs (acetylsalicylic acid, carbasalate calcium, clopidogrel, or dipyridamole) or no antithrombotic treatment at all, at the discretion of the treating physician. Details about the allowed treatments in both groups are shown in Table 2. Treatment will start immediately after randomisation and will continue for the duration of the study. If during the course of a patient’s participation in this study the treating physician feels that a particular antithrombotic drug is clearly indicated or contra-indicated, the choice of the antithrombotic drug may be changed.

**Table 2:** Possible treatments in both trial arms

Arm 1: apixaban	Arm 2: avoid anticoagulation
Apixaban 5 mg twice daily	No antithrombotic treatment
Apixaban 2.5 mg twice daily^a^	Acetylsalicylic acid 80 mg once daily
	Carbasalate calcium 100 mg once daily
	Clopidogrel 75 mg once daily
	Acetylsalicylic acid 80 mg once daily and dipyridamole 200 mg twice daily
	Carbasalate calcium 100 mg once daily and dipyridamole 200 mg twice daily

^a^Reduced dose: the dose will be reduced if 2 of the 3 following criteria are met: age ≥ 80 years, body weight ≤ 60 kg or serum creatinine ≥133 μmol. If the creatinine clearance is below 30 ml per minute, the dose will also be reduced

Patients in both treatment groups will be treated according to the relevant guidelines for the prevention of stroke or systemic embolism.

### Study parameters

We will collect the following parameters: 1) information about the index ICH, including date, National Institutes of Health Stroke Scale score [46], location and volume of the ICH assessed on computed tomography (CT) or magnetic resonance imaging (MRI), antithrombotic medication and INR; 2) previous medical history, including cardiovascular risk factors and previous cardiovascular events, and all variables comprising the CHA_2_DS_2−_VASc [8] and HAS-BLED [47] scores; 3) blood levels of haemoglobin, liver enzymes, and creatinine, and calculated glomerular filtration rate; 4) at the time of randomisation: the score on the mRS, blood pressure, and the Informant Questionnaire on Cognitive Decline in the Elderly (IQCODE), to screen for cognitive decline prior to the index ICH [48].

### Study procedures

After written informed consent will have been obtained, the patient will be randomised. We will collect imaging data obtained in routine clinical care for central reading. At inclusion, the IQCODE will be administered to a person who is close to the patient (partner, family member or friend).

After randomisation, the patient will receive written information related to the assigned treatment to maximise treatment adherence, consisting of a patient alert card for this trial in general and a specific patient alert card for apixaban users. The investigator will send a letter to the general practitioner that will mention inclusion in this trial.

**Fig. 2:**
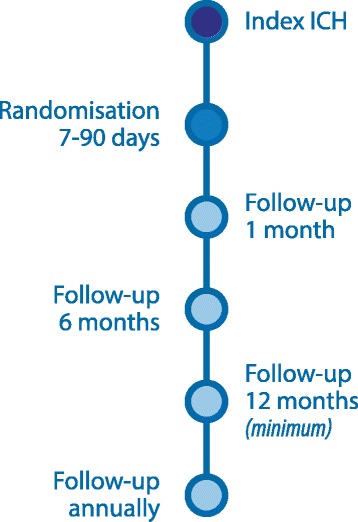
Flowchart of study procedures

At 1 (±7 days), 6 (±14 days), and 12 (±28 days) months and subsequently every 12 months (±28 days), follow-up visits will be scheduled (Fig. 2).

The treating physician or his representative will perform each follow-up visit. Patients will be asked to fill out the Modified Morisky Scale [49] for treatment adherence. Blood pressure will be measured. Disability will be measured with the mRS. Patients will be questioned about the occurrence of outcome events or (other) serious adverse events (SAEs) in the preceding period.

### Study outcomes

The primary outcome is the combination of vascular death or non-fatal stroke (cerebral infarction, ICH, or subarachnoid haemorrhage) during follow-up. Secondary outcomes will be: vascular death, death from any cause, all stroke, ischaemic stroke, ICH, other major extracranial haemorrhage, any intracranial haemorrhage other than ICHd, systemic embolism, myocardial infarction, and functional outcome as assessed with the score on the mRS at 6 and 12 months; annually thereafter; and at the end of the study. Definitions of the clinical events comprising the study outcomes are shown in Table 3.

**Table 3:** Event definitions

Ischaemic stroke	Clinical evidence of the sudden onset of a new neurological deficit, or an increase in an existing deficit, persisting for more than 24 hours, without evidence of a intracerebral haemorrhage on a CT or MRI scan or at post-mortem investigation
Intracerebral haemorrhage	Clinical evidence of the sudden onset of a new neurological deficit, or an increase in an existing deficit, persisting for more than 24 hours, with a corresponding intracerebral haemorrhage on a CT or MRI scan or at post-mortem investigation
Unclassified stroke	Clinical evidence of the sudden onset of a new neurological deficit, or an increase in an existing deficit, persisting for more than 24 hours, without imaging or post-mortem investigations performed
Subarachnoid haemorrhage	Subarachnoid haemorrhage (SAH) demonstrated by CT, lumbar puncture, or at post-mortem investigation
Myocardial infarction	Evidence of myocardial necrosis in a clinical setting consistent with acute myocardial ischemia. Under these conditions any one of the following criteria meets the diagnosis for MI [51]:
	● Detection of a rise and/or fall of cardiac biomarker values (preferably cardiac troponin) with at least one value above the 99th percentile upper reference limit and with at least one of the following:
	○ Symptoms of ischemia
	○ New or presumed new significant ST-segment-T wave changes or new left bundle branch block (LBBB)
	○ Development of pathological Q waves in the ECG
	○ Imaging evidence of new loss of viable myocardium or new regional wall motion abnormality
	○ Identification of an intracoronary thrombus by angiography or autopsy
	● Cardiac death with symptoms suggestive of myocardial ischemia and presumed new ischemic ECG changes or new LBBB, but death occurred before cardiac biomarkers
Vascular death	Death from cerebral infarction; intracerebral, subarachnoid, epidural, or subdural haemorrhage; unclassified stroke; myocardial infarction; extracranial haemorrhage; or systemic embolism, fatal arterial or gastric bleeding, terminal heart failure, fatal pulmonary embolism, and sudden death, defined as death within 1 hour after onset of symptoms
Major extracranial haemorrhage	Major extracranial bleeding will be defined using the ISTH criteria [52];
	1) Fatal bleeding, and/or
	2) Symptomatic bleeding in a critical area or organ, such as intraspinal, intraocular, retroperitoneal, intra-articular or pericardial, or intramuscular with compartment syndrome, and/or
	3) Bleeding causing a fall in haemoglobin level of 1.24 mmol L^−1^ or more, or leading to transfusion of 2 or more units of whole blood or red cells
Clinically relevant non-major bleeding	Clinically relevant non-major bleeding will be defined as acute clinically overt bleeding that does not satisfy additional criteria required for the bleeding event to be defined as a major bleeding event and meets at least one of the following criteria [53]:
	● Hospital admission for bleeding
	● Physician-guided medical or surgical treatment for bleeding
	● Change in antithrombotic (anticoagulant or antiplatelet) therapy
Intracranial haemorrhage	Intracerebral haemorrhage (see above), SAH (see above), subdural haemorrhage: evidence of a subdural haematoma on a CT or MRI scan or at post-mortem investigations; epidural haematoma: evidence of an epidural haematoma on a CT or MRI scan or at post-mortem investigations
Systemic embolism	The diagnosis of systemic embolism requires a clinical history consistent with an acute loss of blood flow to a peripheral artery (or arteries) supported by evidence of embolism from surgical specimens, post-mortem investigations, angiography, vascular imaging, or other objective testing

*CT* computed tomography, *ECG* electrocardiogram, *MRI* magnetic resonance imaging

### Outcome adjudication

An outcome adjudication committee will adjudicate new outcomes on a regular interval. This committee will consist of two experienced vascular neurologists and an experienced cardiologist. The committee will receive outcome information blinded to patient identifiers and treatment allocation.

### Sample size estimation

For the population under study, there are no reliable estimates of the occurrence of the primary outcome for each of the tested treatments, and the main aim of this study is, therefore, to obtain such estimates to inform the design of a phase III clinical trial. Inclusion of a total of 50 patients in each of the treatment arms during the first 30 months of the study and 1 final year of follow-up will result in about 100 patient-years of follow-up in each treatment arm. Ten primary outcome events in 100 patient-years of follow-up will yield a 95 % CI of 4.9 to 17.6. This estimate will not only be more reliable because selection bias will not play a major role in this phase II trial, but also be more precise in comparison with the previous retrospective cohort studies.

### Statistical analysis

The primary analysis will be based on the intention-to-treat principle. As a secondary analysis, we will perform a per-protocol analysis for the primary study outcome. Before the end of the study, a final statistical analysis plan will be completed.

The occurrence of the primary outcome (occurrence of vascular death or a non-fatal stroke) in each of the 2 treatment groups will be expressed as an annual event rate with a 95 % CI. The occurrence of the primary outcomes between the 2 treatment groups will be reported in terms of the hazard ratio with corresponding 95 % CIs, calculated with the Cox proportional hazard model.

The effect of the allocated treatment on the various other outcome events and SAEs will be assessed in the same fashion as the primary study parameter.

We will dichotomise the mRS scores at follow-up into 3 to 6 (poor outcome) and 0 to 2 (good outcome). The effect of the treatment on the mRS will be analysed using risk ratios with a corresponding 95 % CI.

We will adjust the crude hazard ratios and risk ratios for possible baseline incomparability given the size of the study.

### Interim analyses

Interim analyses on safety and efficacy will be performed by the Data Safety and Monitoring Board (DSMB) according to the DSMB charter. These analyses are performed after 50, 100, and 150 patient-years of follow-up, and ad hoc as needed.

The interim analyses on both safety and efficacy will be performed on the primary outcome: the occurrence of vascular death or non-fatal stroke. This combined outcome consists of both the main efficacy outcome (ischaemic stroke) events as well as the main outcomes for harm (ICH, fatal vascular event).

The DSMB will compare both treatment arms using a Poisson’s test (Conditional Test) with two-sided testing. For all interim analyses of the primary outcome, a boundary of *P* < 0.01 will be used for any recommendation to terminate the trial.

### Subject withdrawal

Subjects can leave the study at any time for any reason if they wish to do so without any consequences. The investigator or the treating physician can decide to withdraw a subject from the study for urgent medical reasons. Subjects will not be replaced after withdrawal. In patients who discontinue their allocated treatment, this event will be recorded, including the reason for discontinuation and the new treatment strategy. After discontinuation of the study treatment, follow-up will be carried out as planned.

### Data Safety and Monitoring Board

An independent Data Safety Monitoring Board (DSMB), consisting of a neurologist, a cardiologist, and a biostatistician, monitors the safety and efficacy of the study. See Additional file 1 for the DSMB charter.

### Monitoring

This study has a moderate risk based on the risk classification of the Dutch Federation of University Medical Centres [50], and will be monitored accordingly. The monitoring plan is attached as Additional file 2.

### Safety reporting

Adverse events are defined as any undesirable experience occurring to a subject during the study, whether or not considered related to the allocated treatment. All adverse events reported spontaneously by the subject or observed by the investigator or his staff will be recorded in the medical record on site. Only (suspected) thromboembolic or haemorrhagic adverse events and all SAEs will be reported to the sponsor.

A SAE is any untoward medical occurrence or effect that at any dose:

- results in death
- is life threatening (at the time of the event)
- requires hospitalisation or prolongation of existing inpatients’ hospitalisation
- results in persistent or significant disability or incapacity
- is a congenital anomaly or birth defect

Any other important medical event that may not result in death, be life threatening, or require hospitalisation, may be considered a serious adverse experience when, based upon appropriate medical judgement, the event may jeopardise the subject or may require an intervention to prevent one of the outcomes listed above.

Patients, their partners or families, as well as their general practitioner are requested to report any possible SAE as soon as possible to the local investigator. If the local investigator is notified of a possible SAE or if the local investigator or his staff detect a possible SAE themselves, the local investigator will assess the severity of the adverse event using the criteria described above.

A local investigator will inform the coordinating investigators within 24 hours after he has first knowledge of the event by Email and by filling out the provided SAE form in the electronic data capture platform. All possible SAEs will also be recorded in the patients’ medical record at the site of the local investigator.

The coordinating investigators will report any SAEs to the Medical Research Ethics Committee, pursuant to the Dutch Medical Research Involving Human Subjects Act.

## Informed consent

The local investigator will recruit patients. The treating physician will ask the patient’s or his proxy’s permission to inform the local investigator.

The patient will be informed in person and receive the patient information letter and informed consent form. The first contact with the local investigator will occur no later than on the 76th day after the ICH. The patient will be offered all the available time he deems necessary to consider his decision, but consent by the patient, or in case of incapacity by the patient’s legal representative, must be obtained before or on day 90 after ICH. If an incapacitated subject regains capacity during the study, he will be asked to provide informed consent at that time.

### Ethical considerations

The study will be conducted according to the principles of the Declaration of Helsinki (Fortaleza, Brazil, October 2013) and in accordance with the Dutch Medical Research Involving Human Subjects Act and other guidelines, regulations and Acts.

The study was approved by the University Medical Center Utrecht Medical Research Ethics Committee on 26 August 2014.

The objective of this study can only be accomplished in patients with AF and a recent ICH during treatment with anticoagulants, as we wish to estimate the annual rates of vascular outcomes in patients with both AF and a recent ICH when treated with apixaban and when anticoagulation is avoided. A large proportion of patients who survived an ICH will be incapacitated due to language or cognitive deficits. The clinical dilemma addressed above equally applies to these patients. The inclusion of (temporarily) incapacitated subjects is needed because patients with the capacity to provide informed consent are likely to differ from those without this capacity with respect to lesion location and size. Results obtained in patients with the capacity to consent can, therefore, not readily be extrapolated to patients without this capacity.

For all antithrombotic drugs, a recent ICH is a relative contra-indication to their use and current clinical evidence on this topic is scarce or non-existent. Physicians currently have to rely on their personal clinical judgment to weigh the benefits and risks in prescribing or withholding any antithrombotic therapy in this group. APDs, DOACs such as apixaban, VKAs, and withholding antithrombotic drugs are all strategies used by clinicians today. In this trial, we will include patients in whom there is equipoise on the optimal antithrombotic strategy.

Both a previous ICH and antithrombotic therapy are risk factors for recurrent ICH. There is a risk of recurrent ICH or other major bleeding for all participants, but this is likely to be higher when treated with apixaban or an APD. Conversely, the risk of ischaemic stroke or other thrombo-embolism is increased in patients in whom antithrombotic therapy is withheld. The risk-benefit ratios of all proposed treatments are uncertain.

Apixaban use is a contra-indication for intravenous thrombolysis for acute ischaemic stroke. Patients using apixaban, therefore, cannot be treated with thrombolysis in case of ischaemic stroke during follow-up. However, the risk of ischaemic stroke in patients treated with apixaban will most likely be lower than in patients without antithrombotic therapy or treated with APD and, therefore, we consider this potential disadvantage of apixaban acceptable.

Aside from the bleeding risk, participants allocated to the use of apixaban or an APD are exposed to other side effects of these drugs. The risks of these other side effects are limited.

Investigators will follow their local protocols regarding the management of bleeding in patients using apixaban. Such protocols are available at each study site.

Because both drugs are currently used in this group of patients without any reliable evidence for their net benefit, and because we only include patients in whom clinical equipoise with regard to the optimal treatment strategy exists, we feel we do not expose participants to a significant additional risk in participating in this study compared with current clinical practice.

### Handling and storage of data and documents

All patient data are collected into the electronic data capture platform OpenClinica.

The subjects will be identified using successive numbers, generated by the randomisation system. The key to the code will be maintained by the local investigators in the Investigator Master File. The coordinating investigators will receive identifying and contact information for each participant. This is to enable the coordinating investigator to retrieve information on outcome events if the local investigator does not have this. Participants consent to this with the informed consent form.

Data will be handled according to the Dutch Personal Data Protection Act, Good Clinical Practice and other relevant regulations.

### Public disclosure and publication

Results of the described project will be disclosed and published in peer-reviewed international scientific journals. If the sponsor and/or the investigators will initiate a phase III trial based on the results of the present study, publication of the results of the present trial may be deferred until the results of the phase III trial can be reported. The participant-level dataset will be made available in a public repository within 5 years after the publication of the primary report of the study.

## Discussion

The APACHE-AF trial is the first trial assessing the safety and efficacy of one of the DOACs – apixaban – in a population at risk for both cerebral ischaemia and bleeding. Data obtained from this trial will be instrumental in designing a phase III clinical trial to address the optimal medical treatment of these patients.

### Trial status

Recruitment commenced on 23 September 2014 and the first patients were included in January 2015.

## Appendix 1

### Local Principal Investigators

Yvo BWEM Roos, Academic Medical Center, Amsterdam; Henk Kerkhoff, Albert Schweitzer Ziekenhuis, Dordrecht; Antonia HCML Schreuder, Atrium MC, Heerlen; Michel JM Remmers, Amphia Ziekenhuis, Breda; Diederik WJ Dippel, Erasmus MC, Rotterdam; H Paul Bienfait, Gelre Ziekenhuizen, Apeldoorn; Marieke JH Wermer, Leiden University Medical Center, Leiden; Julie Staals, Maastricht University Medical Center, Maastricht; Heleen M den Hertog, Medisch Spectrum Twente, Enschede; Ewoud J van Dijk, Radboud University Medical Center, Nijmegen; Jeannette Hofmeijer, Rijnstate Ziekenhuis, Arnhem; Jordie H van Tuijl, Sint Elisabeth Ziekenhuis, Tilburg; Renske M van den Berg-Vos, Sint Lucas Andreas Ziekenhuis, Amsterdam, Gert-Jan Luijckx, University Medical Center Groningen, Groningen; H Bart van der Worp, University Medical Center Utrecht.

### Steering committee

Yvo BWEM Roos, Academic Medical Center, Amsterdam; Henk Kerkhoff, Albert Schweitzer Ziekenhuis, Dordrecht; Antonia HCML Schreuder, Atrium MC, Heerlen Michel JM Remmers, Amphia Ziekenhuis, Breda; Diederik WJ Dippel, Erasmus MC, Rotterdam; H Paul Bienfait, Gelre Ziekenhuizen, Apeldoorn; Marieke JH Wermer, Leiden University Medical Center, Leiden; Julie Staals, Maastricht University Medical Center, Maastricht; Heleen M den Hertog, Medisch Spectrum Twente, Enschede; Ewoud J van Dijk, Catharina JM Klijn, Radboud University Medical Center, Nijmegen; Jeannette Hofmeijer, Rijnstate Ziekenhuis, Arnhem; Jordie H van Tuijl, Sint Elisabeth Ziekenhuis, Tilburg; Renske M van den Berg-Vos, Sint Lucas Andreas Ziekenhuis, Amsterdam, Isabelle C van Gelder, Gert-Jan Luijckx, University Medical Center Groningen, Groningen; Ale Algra, L Jaap Kappelle, Koen M. van Nieuwenhuizen, Gabriel JE Rinkel, Rogier EG Schutgens, H Bart van der Worp, University Medical Center Utrecht.

### Executive committee

Catharina JM Klijn, Radboud University Medical Center, Nijmegen; and University Medical Center Utrecht, Utrecht; Koen M van Nieuwenhuizen and H Bart van der Worp, University Medical Center Utrecht, Utrecht.

### Outcome adjudication committee

L Jaap Kappelle, Gabriel J E Rinkel, and Anton E Tuinenburg, University Medical Center Utrecht, Utrecht.

### Data Safety and Monitoring Board

Peter J Koudstaal (chair) and Hendrikus Boersma, Erasmus MC, Rotterdam and Steven AJ Chamuleau, University Medical Center Utrecht, Utrecht.

## Additional files

Additional file 1:
**DSMB Charter.** This file can be viewed with: Adobe Acrobat Reader (http://www.adobe.com/products/acrobat/readstep.html). (PDF 89 kb) Additional file 2:
**Monitoring Plan.** This file can be viewed with: Adobe Acrobat Reader (http://www.adobe.com/products/acrobat/readstep.html). (PDF 52 kb)

## Abbreviations

AF: atrial fibrillation
APACHE-AF: Apixaban versus Antiplatelet drugs or no antithrombotic drugs after intraCerebral HaEmorrhage under anticoagulation for Atrial Fibrillation
APD: antiplatelet drug
ARISTOTLE: Apixaban for Reduction in Stroke and Other Thromboembolic Events in Atrial Fibrillation
AVERROES: Apixaban Versus Acetylsalicylic Acid to Prevent Stroke in Atrial Fibrillation Patients Who Have Failed or Are Unsuitable for Vitamin K Antagonist Treatment
CI: confidence interval
CT: computed tomography
DOAC: direct oral anticoagulant (e.g. apixaban, dabigatran, edoxaban and rivaroxaban)
DSMB: Data Safety Monitoring Board
ICH: intracerebral haemorrhage
INR: international normalised ratio
IQCODE: Informant Questionnaire on Cognitive Decline in the Elderly
ISTH: International Society on Thrombosis and Haemostasis
MI: myocardial infarction
MRI: magnetic resonance imaging
mRS: modified Rankin Scale
OAC: oral anticoagulants
OAC-ICH: oral anticoagulant-associated intracerebral haemorrhage
SAE: serious adverse event
SAH: subarachnoid haemorrhage
TTR: time in therapeutic range (i.e. therapeutic INR when using VKAs)
VKA: vitamin K antagonist (e.g. acenocoumarol, phenprocoumon or warfarin)
