# Supplementary material for: Apixaban versus Antiplatelet drugs or no antithrombotic drugs after anticoagulation-associated intraCerebral HaEmorrhage in patients with Atrial Fibrillation (APACHE-AF): study protocol for a randomised controlled trial
Source: Trials. 2015 Sep 4;16:393. doi: 10.1186/s13063-015-0898-4 (PMC4560912; doi:10.1186/s13063-015-0898-4)
Supplement: Additional file 1: — DSMB Charter. This file can be viewed with: Adobe Acrobat Reader (http://www.adobe.com/products/acrobat/readstep.html). (PDF 89 kb) [file 13063_2015_898_MOESM1_ESM.pdf]

DSMB Charter

NL47761.041.14

APACHE-AF: Apixaban versus Antiplatelet drugs or no antithrombotic drugs after Cerebral HaEmorrhage under anticoagulation for Atrial Fibrillation.

Authors:
K.M. van Nieuwenhuizen
C.J.M. Klijn
H.B. van der Worp

Version: 1.0
Status: Final
Date: June 6, 2014

1. Introduction

1.1 Study identification

Study title:	Apixaban versus antiplatelet drugs or no antithrombotic drugs after anticoagulation-associated intracerebral haemorrhage in patients with atrial fibrillation. A randomised phase II clinical trial.
Short title:	APACHE-AF: Apixaban versus Antiplatelet drugs or no antithrombotic drugs after Cerebral HaEmorrhage under anticoagulation for Atrial Fibrillation.
ABR number:	NL47761.041.14
EudraCT number:	2014-000112-33
Netherlands Trial register:	NTR4526

1.2 Trial description

This study compares treatment with apixaban vs. treatment with antiplatelet drugs or no antithrombotic treatment in patients who had an intracerebral haemorrhage while using oral anticoagulation because of atrial fibrillation.

1.2.1 Interventions

First study arm: apixaban 5 mg twice daily
Second study arm: one of five antiplatelet drugs treatments or no antithrombotic treatment, at the discretion of the treating physician.

1.2.2 Objectives

The primary objective is to obtain reliable estimates of the rates of vascular death or non-fatal stroke in patients with atrial fibrillation and a recent anticoagulation-associated intracerebral haemorrhage who are treated with apixaban versus those who are treated with an APD or no antithrombotic drug.

The secondary objective is to compare the rates of all-cause death, disabling stroke, major haemorrhage, systemic embolism and functional outcome between patients treated with apixaban and those who are treated with an APD or no antithrombotic drug.

1.3 Scope of this document

The purpose of this document is to describe the roles and responsibilities of the independent DSMB for the APACHE-AF trial, including the timing of meetings, methods of providing

information to and from the DSMB, frequency and format of meetings, statistical issues and relationships with other boards or committees.

2. Roles and responsibilities

2.1 Aims of the board

To safeguard the interests of trial participants, assess the safety and efficacy of the interventions during the trial, and monitor the overall conduct of the clinical trial. The DSMB adheres to the principles of the DAMOCLES statement.[1]

2.2 Terms of reference

The DSMB should receive and review the progress and accruing data of this trial and provide advice on the conduct of the trial to the Coordinating Investigators.

The DSMB should inform the Coordinating Investigators if, in their view:

(i) the results are likely to convince a broad range of clinicians, including those supporting the trial and the general clinical community, that one trial arm is clearly indicated or contraindicated; and

(ii) there is a reasonable expectation that this new evidence would materially influence patient management; or

(iii) the safety of the participants is threatened; or

(iv) it becomes evident that no clear result will be obtained.

2.3 DSMB roles and responsibilities

The main objective of this DSMB is to:

1. safeguard the interests of patients included in the trial.

The responsibilities of the DSMB will be to:

1. evaluate aggregate and individual subject data related to safety;
2. evaluate date integrity and overall conduct of the trial based on reports by the study monitor;
3. evaluate recruitment figures and losses to follow-up;
4. evaluate evidence for treatment differences in the main efficacy outcomes;
5. evaluate evidence for treatment harm (e.g. SAEs, deaths);
6. decide whether to recommend that the trial continues to recruit participants or whether recruitment should be terminated;
7. advise on protocol modifications suggested by investigators or the sponsor;

8. consider the need for additional unscheduled reviews of study data;
9. evaluate compliance with previous DSMB recommendations;
10. consider the ethical implications of any recommendation made by the DSMB;
11. assess the impact and relevance of external evidence.

In order to fulfil its responsibilities, the DSMB will:

1. operate according to the procedures described in this charter;
2. meet periodically.

2.4 Sponsor Roles and Responsibilities

The Sponsor will

2. assure the proper conduct of the study;
3. ensure timely provision of relevant trial data to the DSMB, and ensure that these are accurate;
4. compile and report SAEs to the DSMB;
5. promptly report potential safety concern(s) to the DSMB;
6. prepare summary reports of relevant data to the DSMB This may include analyses not otherwise outlined in this charter if deemed necessary by the Sponsor;
7. forward reports from the study monitor to the DSMB;
8. inform the DSMB on relevant external data;
9. communicate with regulatory authorities, METC, and investigators, as necessary;
10. facilitate the regular DSMB meetings by providing means of communication;
11. reimburse travel costs to DSMB members, if applicable (see also 3.3).

3. Membership

This DSMB will consist of 3 members as listed in Appendix A. One member will be a neurologist, one a cardiologist and one a clinical epidemiologist or statistician.

The Sponsor will appoint the chairman. The chairman will have substantial experience in serving as a DSMB member and will be appointed by the Sponsor.

3.1 Member qualifications

As characteristic qualifications, members will:

- Work professionally and meet qualifications for their respective professions.
- Have experience in conducting clinical trials
- Comply with accepted practices of their respective professions.

- Comply with the conflict of interest policies specified by the Sponsor
- Not have serious scientific, financial, personal, or other conflicts of interest related to the conduct, outcome, or impact of the study according to the guidelines specified below and complete a conflict of interest form (appendix B) before their membership begins and if any changes in potential or real conflict of interests occur.
- Be independent from the METC, regulatory agencies, coordinating investigator, site investigator, or any other capacity related to trial operations. Colleagues at their work place may be investigators in the trial.

3.2 Replacement

DSMB members will be appointed for the duration of the trial. If a DSMB member is unable to continue his membership, the Sponsor will appoint a replacement.

3.3 Reimbursement

DSMB members will be reimbursed for travel costs. No other reimbursement will be paid.

4. Emerging safety information

For the entire duration of the trial, the Coordinating Investigators will send a report for each occurrence of the primary outcome and any serious adverse event (SAE) or suspected unexpected serious adverse reactions (SUSAR) to the DSMB, within 21 days after the coordinating investigator has first knowledge of the event. This report includes the treatment allocation.

5. Meetings

5.1 Meeting schedule

This DSMB will meet by telephone or face-to-face after 50, 100, and 150 patient-years of follow-up, but at least yearly after the start of the study.

5.2 Meeting organization

This is an open clinical trial with blinded outcome assessment. The investigators will remain blinded to the results of the study until all outcomes have been adjudicated and locked after the final follow-up of the last patient. The DSMB will not be blinded.

All DSMB meetings will begin with an open session, which the coordinating investigators will attend. This will be followed by a closed session, only attended by the DSMB members.

Minutes of both sessions will be recorded by a DSMB member appointed by the DSMB chairman.

Two weeks before the meeting, the coordinating investigators, in collaboration with the trial clinical epidemiologist, will provide the DSMB with the necessary data and reports. The coordinating investigators will provide the DSMB with data from other studies if relevant for the present trial.

5.2.1 Open session

During the open part of the DSMB meeting, information relating to recruitment will be presented. Total numbers of events for the primary outcome and other outcomes may be presented, at the discretion of the DSMB.

5.2.2 Closed session

Unblinded study data on outcomes and SAEs will be available to the DSMB during the closed session.

5.3 Communications

The DSMB will send a report of their recommendations and decisions to the coordinating investigators.

5.4 Confidentiality

The DSMB members will ensure the confidentiality of the information received from the coordinating investigators. The minutes of the open sessions will be sent to the coordinating investigators and kept confidential; the DSMB members are not allowed to share any information with others.

6. Decisions

6.1 Possible recommendations

The DSMB can recommend any of the following after a DSMB session:

- No action needed, trial continues as planned
- Early stopping due, for example, to clear evidence from the trial of benefit or harm of a treatment, or external evidence
- Extending recruitment (or extending follow-up
- Protocol changes

6.2 Interim analysis

6.2.1 Primary outcome

The interim analyses on both safety and efficacy will be performed on the primary outcome: the occurrence of vascular death or non-fatal stroke. This combined outcome consists of both the main efficacy outcome (ischaemic stroke) events as well as the main outcomes for harm (intracerebral haemorrhage, fatal vascular event).

The DSMB will compare both treatment arms using a Poisson's test (Conditional Test) with two-sided testing. For all interim analyses of the primary outcome, a boundary of $p < 0.01$ will be used for any recommendation to terminate the trial.

6.3 Decision making

A decision can only be made if all 3 members are present at the meeting. The members will strive to make an unanimous decision, weighing both the results of the interim analysis and the ethical implications of their decision. If an unanimous decision cannot be reached, the members will vote. Each member's vote will weigh equally.

7. Reporting

7.1 Meeting report

The chairman will compose a letter to the Coordinating Investigators within 3 weeks of the DSMB meeting. This letter will be filed in the Study Master File, section L5. The DSMB chair will archive the minutes of each meeting until 15 year after the completion of the trial.

7.2 Disagreement

If the Coordinating Investigators choose to disregard the DSMB recommendation, a meeting will be held between the DSMB (or at least the chairman and statistician) and the Coordinating Investigators. This meeting will be chaired by an external expert, not involved in the study, appointed by the DSMB chairman. A report of this meeting will be sent to the METC of the UMC Utrecht.

8. End of study

8.1 Publication

The Coordinating Investigators will submit the results of the present study to a peer-reviewed journal. Publication can be deferred if the Sponsor and/or Coordinating Investigators will

conduct or participate in a phase III trial based on the results of the present study if the results of this study will be pooled with the phase III study.

The names of the DSMB members and a brief description of the conclusion of their meetings will be reported in the main paper.

8.2 Disclosure

Members will not disclose any information on the results of this study before this study is published or until the Sponsor permits disclosure.

Appendix A

By signing this sheet, the members

- Agree to serve on the DSMB of the present study
- Agree with DSMB charter and will conduct their DSMB tasks accordingly

Prof. P.J. Koudstaal, MD, PhD, neurologist and DSMB chairman

Date Signature

S.A.J. Chamuleau, MD, PhD, cardiologist

Date Signature

Prof. H. Boersma, MSc, PhD, FESC, biostatistician

Date Signature

Appendix B

Potential competing interests of Data Safety Monitoring Board members for APACHE-AF: Apixaban versus Antiplatelet drugs or no antithrombotic drugs after Cerebral HaEmorrhage under anticoagulation for Atrial Fibrillation.

The avoidance of any perception that members of a DSMB may be biased in some fashion is important for the credibility of the decisions made by the DSMB and for the integrity of the trial.

Possible competing interest should be disclosed via Sponsor. In many cases simple disclosure up front should be sufficient. Otherwise, the (potential) DSMB member should remove the conflict or stop participating in the DSMB. Table 1 lists potential competing interests.

Table 1: Potential competing interests

- Stock ownership in any of the commercial companies involved
- Stock transaction in any commercial company involved (if previously holding stock)
- Consulting arrangements with one or more of the producers of the drugs tested;
- Having received speaker's fees from one or more of the producers of the drugs tested;
- Career tied up in a product or technique assessed by trial
- Hands-on participation in the trial
- Involvement in the conduct of the trial
- Emotional involvement in the trial
- Intellectual conflict, e.g. strong prior belief in the trial's experimental arm
- Involvement in regulatory issues relevant to the trial procedures
- Investment (financial or intellectual) in competing products
- Involvement in the publication

Please complete the following section and return to the Sponsor

□ No, I have no competing interests to declare
□ Yes, I have competing interests to declare (please detail below)

Please provide details of any competing interests:

Name: ____________________________

Signed: ___________________________ Date: ______________

9. References

1. Campbell PMK. A proposed charter for clinical trial data monitoring committees: helping them to do their job well. Lancet. 2005;365(9460):711–22.
