# Supplementary material for: Apixaban versus Antiplatelet drugs or no antithrombotic drugs after anticoagulation-associated intraCerebral HaEmorrhage in patients with Atrial Fibrillation (APACHE-AF): study protocol for a randomised controlled trial
Source: Trials. 2015 Sep 4;16:393. doi: 10.1186/s13063-015-0898-4 (PMC4560912; doi:10.1186/s13063-015-0898-4)
Supplement: Additional file 2: — Monitoring Plan. This file can be viewed with: Adobe Acrobat Reader (http://www.adobe.com/products/acrobat/readstep.html). (PDF 52 kb) [file 13063_2015_898_MOESM2_ESM.pdf]

Monitoring plan

NL47761.041.14

APACHE-AF: Apixaban versus Antiplatelet drugs or no antithrombotic drugs after Cerebral HaEmorrhage under anticoagulation for Atrial Fibrillation.

Authors:
K.M. van Nieuwenhuizen
C.J.M. Klijn
H.B. van der Worp

Version: 2.0
Status: Final
Date: January 23, 2015

1. Risk classification

This study has a moderate risk, based on the risk classification of the Dutch Federation of University Medical Centers

2. Execution of monitoring

Monitoring will be performed qualified monitors, not involved in the design or execution of the trial.

3. Frequency of monitoring

Each site will be visited annually if 1 to 5 participants are enrolled. If 6 or more participants are enrolled, each site will be visited biannually. Additionally, each site will have an initiation visit and a visit one month after inclusion of the first patient. Each site with at least one patient enrolled will have a close-out visit at the end of the study. A close-out visit can be combined with the last annual or biannual visit.

4. Source data verification

The following monitoring will be performed on source data:

- 100% of the informed consent forms for existence and correctness
- 100% of the inclusion and exclusion criteria for the first 5 participants in each site. Subsequently, the inclusion and exclusion criteria of 25% of the participants will be verified. If an erroneous inclusion is found, all files in that site will be verified.
- 25% of the prescription of the allocated treatment.
- 25% of the occurrence of follow-up visits or contacts.
- 25% verification of the correct SAE, SAR and SUSAR reporting procedure.
- 25% of participant records will be checked for missed SAEs, SARs or SUSARs. If a missed SAE, SAR or SUSAR is found, all files in that site will be verified.
- 25% of participants records will be checked for missed primary outcomes, as specified in C1 Research Protocol chapter 7.1.1.
- 10% of participants records will be checked for missed secondary outcomes, as specified in C1 Research Protocol chapter 7.1.2

5. General monitor tasks

- The monitor will report the speed of inclusion and dropout rate per site at each visit.
- For each site the presence and completeness of the Investigator Site File and for the Sponsor the Study Master File will be verified.

- For each site the presence of study procedures will be verified. The compliance and competence of study personnel will be verified.

6. Reporting

The monitor will provide the principal investigator with a written report after every visit to a site. This report will be filed by the principal investigator. The local investigator for each site will receive a written summary of the monitoring activities and the monitoring findings.

Every monitor visit report will contain:

- A summary of the items assessed by the monitor
- A general description of quality
- A list of all the important findings, anomalies and shortcomings
- An overview of all pending actions and recommendations to ensure protocol adherence.
- An overall conclusion

The Sponsor’s coordinating investigators will receive the receive the original initiation visit and close-out visit reports, the local investigator will receive a copy of these documents. If relevant, other communications regarding the study will be recorded.
